# Regulation of oxidative phosphorylation is different in electrically- and cortically-stimulated skeletal muscle

**DOI:** 10.1371/journal.pone.0195620

**Published:** 2018-04-26

**Authors:** Bernard Korzeniewski

**Affiliations:** Faculty of Biochemistry, Biophysics and Biotechnology, Jagiellonian University, Kraków, Poland; University of Wisconsin Madison, UNITED STATES

## Abstract

A computer model of the skeletal muscle bioenergetic system was used to study the regulation of oxidative phosphorylation (OXPHOS) in electrically-stimulated and cortically-stimulated skeletal muscle. Two types of the dependence of the intensity of each-step activation (ESA) of OXPHOS complexes on ATP usage activity were tested: power-type dependence and saturating-type dependence. The dependence of muscle oxygen consumption ($\dot{V}O_{2}$), phosphocreatine (PCr), cytosolic ADP, ATP, inorganic phosphate (P_i_), pH and τ_p_ (characteristic transition time) of the principal component of the muscle $\dot{V}O_{2}$ on-kinetics on the ATP usage activity was simulated for both types of the ESA intensity-ATP usage activity dependence. Computer simulations involving the power-type dependence predict system properties that agree well with experimental data for electrically-stimulated muscle. On the other hand, model predictions for the saturating-type dependence in the presence of the ‘additional’ ATP usage (postulated previously to underlie the slow component of the VO_2_ on-kinetics) reproduce well system properties encountered in human skeletal muscle during voluntary exercise. It is postulated that the difference between the regulation and kinetic properties of the system in electrically- and cortically-stimulated muscle is mostly due to the different muscle fibers recruitment pattern. In the former, all fiber types are recruited in parallel already at low power output (PO) values, while in the latter type I fibers (with higher ESA intensity) are stimulated at low PO values, while type II fibers (especially type II b and IIx fibers) with low ESA intensity are recruited predominantly at high PO values.

## Introduction

Skeletal muscle contraction is driven by hydrolysis of ATP to ADP and P_i_. At rest ATP is needed to sustain such basic processes keeping the muscle fiber alive as protein / RNA synthesis or ion (Na^+^, K^+^, Ca^2+^) circulation across cellular membranes. During exercise, ATP is mainly used by actomyosin-ATPase and Ca^2+^-ATPase (SERCA). The dependence of system variables ($\dot{V}O_{2}$, PCr, cytosolic P_i_, ATP, ADP, pH) on ATP usage activity is of key significance for understanding of the regulation and behavior of the skeletal muscle bioenergetic system during rest-to-work transition.

In a given exercise type the ATP usage activity A_UT_ (relative activation of ATP usage, increase in its rate constant k_UT_ in relation to rest) can be assumed to be linearly proportional to work intensity (power output). The ‘additional’ ATP usage [1] was postulated to be mostly responsible for the slow-component of the $\dot{V}O_{2}$ on kinetics [1–4]. Its inclusion in the computer model gives an excellent agreement of computer simulations with experimental data concerning fluxes and metabolite concentrations during rest-work-recovery transition in skeletal muscle [1]. It appears above the ‘critical’ relative ATP usage activity (related to critical power, CP), and its relative activity is assumed in the present study to be linearly proportional to the difference between the current and the critical relative ATP usage activity, and to increase linearly with time, which again agrees well with experimental data showing an increase in the intensity of the slow component with work intensity [5].

It was postulated that the main mechanism responsible for the regulation of the cell bioenergetic system, especially oxidative phosphorylation (OXPHOS), during work transitions in skeletal muscle, heart and other tissues is the so-called each-step activation (ESA) mechanism, a special case of the broader parallel-activation mechanism. According to ESA, not only ATP usage and NADH supply, but also all OXPHOS complexes (complex I, complex III, complex IV, ATP synthase, ATP/ADP carrier, P_i_ carrier) and glycolysis are directly activated by some cytosolic mechanism predominantly involving cytosolic Ca^2+^ ions and perhaps calmodulin-like protein responsible for protein phosphorylation, during rest-to-work transition in skeletal and heart muscle cells [1,6–10]. In skeletal muscle it is likely that a mixed mechanism (MM) is manifest, in which all OXPHOS complexes are directly activated, but to a smaller extent than ATP usage, and therefore a moderate increase in [ADP] and [P_i_] cooperates with ESA to bring about OXPHOS activation [10]. In intact heart *in vivo* there is no (or small) change in metabolite concentrations during work transitions [11]. Therefore, it was postulated that ESA, directly activating both ATP usage and OXPHOS to the same extent, is essentially the sole mechanism operating in intact heart *in vivo* [12,13].

The ESA, each-step activation intensity A_OX_ (relative direct activation of OXPHOS and NADH supply in relation to rest) determines how many times the activity of OXPHOS and NADH supply (the rate constants of all OXPHOS complexes: k_C1_, k_C3_, k_C4_, k_SN_, k_EX_, k_PI_ and of the NADH supply block: k_DH_) is (are) elevated during rest-to-work transition. In some previous theoretical studies a power-type A_OX_-A_UT_ dependence was assumed as the simplest possibility. According to this dependence, A_OX_ continuously increases as a power function of A_UT_ (ATP usage activity) and never reaches a plateau. Quantitatively, the power-type dependence has the following form: A_OX_ = A_UT_^pOX^, where the power coefficient pOX, equal in most simulations to 0.3–0.5, is the measure of the ESA strength. However, a recent theoretical study [1] demonstrated that the actual A_OX_-A_UT_ dependence is saturating-type in human bilateral knee extension exercise. This means that A_OX_ first increases with A_UT_ increase, but afterwards stabilizes on a constant level. It was estimated [1] that A_OX_, by definition equal to 1 at rest, equals about 5.6 in moderate exercise and 5.2 in heavy / severe exercise. At the same time, the assessed A_UT_ (by definition equal to 1 at rest) was much higher in heavy / severe exercise: 47, than in moderate exercise: 22. Therefore, in this case the ESA, each-step activation intensity A_OX_ does not increase between moderate and heavy / severe work intensity, when A_UT_ (PO) is largely elevated, and the A_OX_-A_UT_ dependence is saturating-type.

It is of course interesting and important for understanding of the functioning of the system how different types of the A_OX_-A_UT_ dependence affect the dependence of system variables, such as muscle $\dot{V}O_{2}$, PCr, cytosolic P_i_, ATP, ADP, pH and the characteristic transition time τ_p_ of the principal component (phase II) of the muscle $\dot{V}O_{2}$ on-kinetics on the ATP usage activity (A_UT_) (and thus work intensity). It seems also interesting how the presence of the ‘additional’ ATP usage above the critical ATP usage activity (related to critical power, CP) affects this dependence.

However, the most important challenge is to test whether the saturating-type *versus* power-type and A_OX_-A_UT_ dependence is able to account for the differences in the kinetic properties of the bioenergetic system in cortically-stimulated (voluntary exercise) *versus* electrically-stimulated skeletal muscle. In other words, it seems very interesting whether the regulation of OXPHOS is different in both sorts of exercise and, if so, what this difference consists in.

It was postulated that ESA tends to be more intensive in oxidative skeletal muscle (fibers), in muscle during voluntary exercise (cortical stimulation) and in intact muscle with physiological blood flow than in glycolytic skeletal muscle (fibers), in electrically-stimulated muscle and in perfused muscle [7]. Changes in metabolite (PCr, Cr, ADP) concentrations and pH during work transitions are much greater in glycolytic muscles (composed mostly of type II fibers) than in oxidative muscles (composed predominantly of type I fibers) (see e.g., [14]), and it was postulated that the main role of ESA is to maintain as good metabolite and pH homeostasis as possible [6–10]. In voluntary exercise (cortically-stimulated muscle), there is a sequential pattern of recruitment of particular muscle fiber types when work intensity increases: oxidative type I fibers with high OXPHOS capacity (and ESA intensity) are recruited mainly (or exclusively) at low PO values, followed by the recruitment of also predominantly oxidative type IIa muscle fibers, and finally of predominantly glycolytic type IIx and IIb muscle fibers when PO approaches its maximum values. This is controlled by neural stimulation of particular motor units [15,16]. In electrically-stimulated muscle different muscle fibers (type I and various type II fibers) are stimulated non-specifically when the stimulation frequency increases, even at lowest stimulation frequencies (ATP usage activities), and the work performed is proportional to the stimulation frequency.

The present study is intended first of all to reveal the differences in the regulation of the bioenergetic system, especially OXPHOS, between the cortically- and electrically-stimulated skeletal muscle during constant-power exercise. The dependence of selected skeletal muscle bioenergetic system variables (muscle $\dot{V}O_{2}$, PCr, cytosolic P_i_, ATP, ADP, pH and τ_p_ of the muscle $\dot{V}O_{2}$ on-kinetics) on ATP usage activity A_UT_ is simulated for three possibilities: 1. Power-type A_OX_-A_UT_ dependence in the absence of the ‘additional’ ATP usage; 2. Saturating-type A_OX_-A_UT_ dependence in the absence of the ‘additional’ ATP usage; and 3. Saturating-type A_OX_-A_UT_ dependence in the presence of the ‘additional’ ATP usage. It is hypothesized that the power-type A_OX_-A_UT_ dependence is able to account for the kinetic behavior of the system encountered in electrically-stimulated muscle, while the saturating-type A_OX_-A_UT_ dependence in the presence of the ‘additional’ ATP usage can explain the system properties in cortically-stimulated muscle (voluntary exercise in humans). Confrontation of computer simulations with various experimental data supports this hypothesis. It is postulated that the different regulation of OXPHOS in electrically- and cortically-stimulated skeletal muscle results from different patterns of muscle fiber recruitment when A_UT_ increases. In electrically-stimulated muscle all fiber types are recruited in parallel already at lowest stimulation frequencies. On the other hand, in cortically-stimulated muscle during voluntary exercise most of oxidative type I fibers (that are postulated to have high ESA intensity) are recruited first, at low and moderate work intensities, followed by recruitment of also predominantly oxidative type IIa fibers, and finally, at highest work intensities, of predominantly glycolytic type IIx and IIb fibers (that are postulated to have low ESA intensity).

## Theoretical methods

### Computer model

The computer model of OXPHOS and the entire bioenergetic system in intact skeletal muscle [17,18] was used in the simulations carried out in the present study. The model was recently modified by replacing first-order inhibition of glycolysis by protons with third-order inhibition [1]. This model comprises explicitly NADH supply block (TCA cycle, fatty-acid β-oxidation, MAS *etc*.), particular OXPHOS complexes (complex I, complex III, complex IV, ATP synthase, ATP/ADP carrier, P_i_ carrier), proton leak through the inner mitochondrial membrane, glycolysis (aerobic and anaerobic), ATP usage, creatine kinase (CK) and proton efflux/influx to/from blood. The complete model description of particular model versions is located on the web site: http://awe.mol.uj.edu.pl/~benio/.

### ESA intensity-ATP usage activity dependence

The relative activity of ATP usage (relative increase in its rate constant k_UT_ in relation to rest) A_UT_ between 1 (rest) and 100 (maximum A_UT_) was used in different sets of subsequent computer simulations. Two types of dependencies of the intensity of each-step activation (ESA) of OXPHOS (and NADH supply) (relative increase of the rate constants of all OXPHOS complexes and NADH supply block in relation to rest) A_OX_ on A_UT_ were tested: power-type dependence and saturating-type dependence.

The power-type dependence, used (assumed as the simplest possibility) in some previous theoretical studies, was described by the following equation:

$$A_{OX}=A_{UT}^{p_{OX}}$$ (1)

where A_OX_ (ESA, each-step activation intensity) is the relative OXPHOS (+ NADH supply) activity (activation in relation to rest), A_UT_ is the relative ATP usage activity (activation in relation to rest) and the power coefficient p_OX_ = 0.45 is used in the present study; p_OX_ is a measure of ESA strength (p_OX_ = 0.45 means a relatively strong ESA).

The saturating-type dependence, introduced for the first time in the present study on the basis of the data extracted from experimental studies [1], was described by the following equation:

$$A_{OX}={1+A}_{OXmax}\left(\frac{A_{UT}-1}{(A_{UT}-1)+K_{A_{UT}}}\right)$$ (2)

where A_OX_ (ESA, each-step activation intensity) is the relative OXPHOS (+ NADH supply) activity (activation in relation to rest), A_UT_ is the relative ATP usage activity (activation in relation to rest), A_OXmax_ = 5 is the maximum A_OX_—1 (thus maximum A_OX_ = 6) and K_AUT_ = 5 is the ‘half-saturating’ A_UT_ value for the increase in A_OX_. The values of A_OXmax_ and K_AUT_ were chosen in order to reproduce the experimental data (see below). The values of A_OX_ and A_UT_ for rest, moderate exercise and heavy / severe exercise that served to construct this equation were taken from [1].

**Fig 1:**
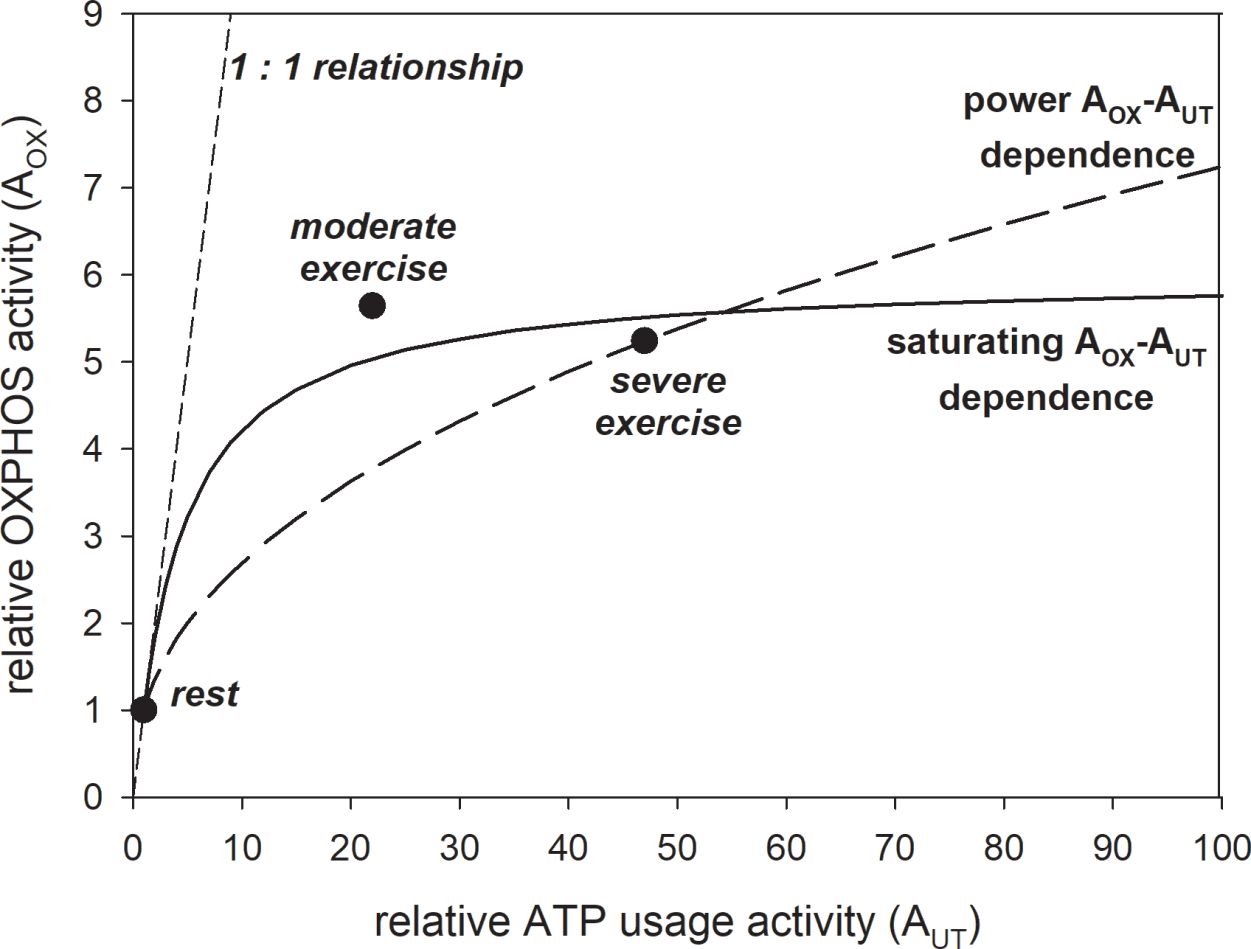
Power-type vs. saturating-type A_OX_ (ESA, each-step activation intensity)-A_UT_ (relative ATP usage activity) dependence. Simulated power-type and saturating-type A_OX_-A_UT_ dependences (lines) are compared with the values of A_OX_ and A_UT_ extracted from experimental data for rest, moderate exercise and severe exercise (points) [1]. The power-type dependence, described by Eq 1, is postulated to be present in electrically-stimulated muscle, while the saturating-type dependence, described by Eq 2, is postulated to be present during voluntary exercise (cortically-stimulated muscle).

Both dependencies are presented in Fig 1. One can see that the saturating-type dependence fits better than the power-type dependence to the relationship between the relative activity of OXPHOS (ESA, each-step activation intensity) A_OX_ and the relative activity of ATP usage A_UT_ for rest, moderate exercise and severe exercise extracted from experimental data concerning voluntary constant-power knee-extension exercise in humans (see [1]).

The dependence between the relative glycolysis activity A_GL_ and A_UT_ was described in both cases by a power-type dependence:

$$A_{GL}=A_{UT}^{p_{GL}}$$ (3)

where A_GL_ is the relative glycolysis activation (relative increase in the rate constant of glycolysis k_GL_ in relation to rest), A_UT_ is relative ATP usage activity and the power coefficient p_GL_ is a measure of ESA strength for glycolysis. p_GL_ = 0.8 for power-type A_OX_-A_UT_ dependence and p_GL_ = 0.55 for saturating-type A_OX_-A_UT_ dependence. These values were fitted in order to obtain reasonable values of cytosolic pH at high A_UT_ (relative ATP usage activity) values (about 6.75, drop by 0.25 in relation to rest, at maximum A_UT_ = 100 used in the present study). The value of p_GL_ is different for power-type and saturating-type A_OX_ (ESA intensity)-A_UT_ dependence, as A_OX_ at high A_UT_ values is different in these cases. This power-type A_GL_ (relative glycolysis activity)-A_UT_ dependence allows for significant (anaerobic) glycolysis stimulation at high work intensities (A_UT_ values) that takes place in real muscles under these conditions.

### ‘Additional’ ATP usage kinetics

It was postulated that the ‘additional’ ATP usage [1], underlying the slow component of the $\dot{V}O_{2}$ on-kinetics, appears when the relative ATP usage activity A_UT_ exceeds the critical relative ATP usage activity (related to critical power, see Discussion) [1,4]. The absolute ‘additional’ ATP usage activity (rate in mM min^-1^) is described in the present study by the following equation:

$$v_{UTadd}=k_{UTadd}{∙v}_{UT}∙(A_{UT}-A_{UTcrit})∙t_{exerc}$$ (4)

where v_UTadd_ is the absolute ‘additional’ ATP usage activity (in mM min^-1^), k_UTadd_ is the ‘rate constant’ of the increase in the absolute ‘additional’ ATP usage in time (in min^-1^), A_UT_ is the relative ATP usage activity (activation in relation to rest) (unitless), A_UTcrit_ is the critical relative ATP usage activity (unitless) and t_exerc_ is time (min) after the onset of exercise. This equation means that v_UTadd_ increases both with A_UT_ above A_UTcrit_ and with time after the onset of exercise. The linear increase in the ‘additional’ ATP usage v_UTadd_ in time gives an excellent agreement of model predictions with experimental data [1]. The increase of v_UTadd_ in time is related to the increase in the slow component of the $\dot{V}O_{2}$ on-kinetics in time [1,4], while the increase in the ‘additional’ ATP usage v_UTadd_ with A_UT_ above the critical ATP usage A_UTcrit_ is due to the fact that the extent of the slow component increases with PO [5,19]. In the simulations carried out in the present study it is assumed that A_UTcrit_ = 50 (a half of the maximum A_UT_ = 100) and k_UTadd_ = 0.0008 min^-1^. The first assumption is justified by the observation [20] that the non-linearity in the $\dot{V}O_{2}$-PO dependence, beginning at PO value where the ‘additional’ ATP usage appears, started at 35–65% of the maximum power output (PO_max_).

The total absolute ATP usage activity A_UTtot_ (in mM min^-1^) is equal to the sum of the normal and ‘additional’ absolute ATP usage activity:

$$v_{UTtot}=v_{UT}+v_{UTadd}$$ (5)

### Work transitions

During rest-to-work transition in skeletal muscle the ATP usage was activated A_UT_ times (the rate constant of ATP usage k_UT_ was increases A_UT_ times). At the same time OXPHOS and NADH supply were activated A_OX_ times (the rate constants of complex I: k_C1_, complex III: k_C3_, complex IV: k_C4_, ATP synthase: k_SN_, ATP/ADP carrier: k_EX_, P_i_ carrier: k_PI_ and NADH supply: k_DH_ were increased A_OX_ times). Glycolysis was activated A_GL_ times (the rate constant of glycolysis k_GL_ was increased A_GL_ times).

During the opposite transition (work-to rest transition) the resting ATP usage activity, OXPHOS activity, NADH supply activity and glycolysis activity were restored.

An instantaneous increase of the ATP usage activity (increase in k_UT_) during on-transient and decrease of the ATP usage activity (decrease in k_UT_) during off-transient was applied in computer simulations. On the other hand, some (although relatively very short, see below) delay in the increase of the activity of OXPHOS (and NADH supply) and glycolysis during on-transient and in the decrease of the activity of these processes during off-transient was assumed in computer simulations. The time-dependent activation after the onset of elevated work was described by the following equation:

$$m_{X}=A_{X}-\left(A_{X}-1\right)\cdot e^{-t/t{\left(ON\right)}_{X}}$$ (6)

where X is OX (oxidative phosphorylation + NADH supply) or GL (glycolysis), m_X_ is the current (at time t) relative activation of X (multiplicity of the rest value(s) of its rate constant(s)), t(ON)_X_ is the characteristic activation time of X, and t is the time after the onset of elevated work (rest-to-work transition). The time-dependent inactivation (decay) after the termination of muscle work was described by the following equation:

$$m_{X}=1+\left(A_{X}-1\right)\cdot e^{-t/t{\left(OFF\right)}_{X}}$$ (7)

where X is OX (oxidative phosphorylation + NADH supply) or GL (glycolysis), m_X_ is the current (at time t) relative activation of X (multiplicity of the rest value(s) of its rate constant(s)), t(OFF)_X_ is the characteristic inactivation time of X and t is the time after the termination of elevated work (work-to-rest transition).

In the present study the following characteristic transition times were used: t(ON)_OX_ = 3 s, t(ON)_GL_ = 6 s, t(OFF)_OX_ = 120 s, t(OFF)_GL_ = 1 s (see [1]). t(ON)_OX_ was estimated for 11 s in electrically-stimulated muscle [21]. It was shown that $\dot{V}O_{2}$ starts to increase almost instantaneously after the onset of exercise in electrically-stimulated muscle [22].

**Fig 2:**
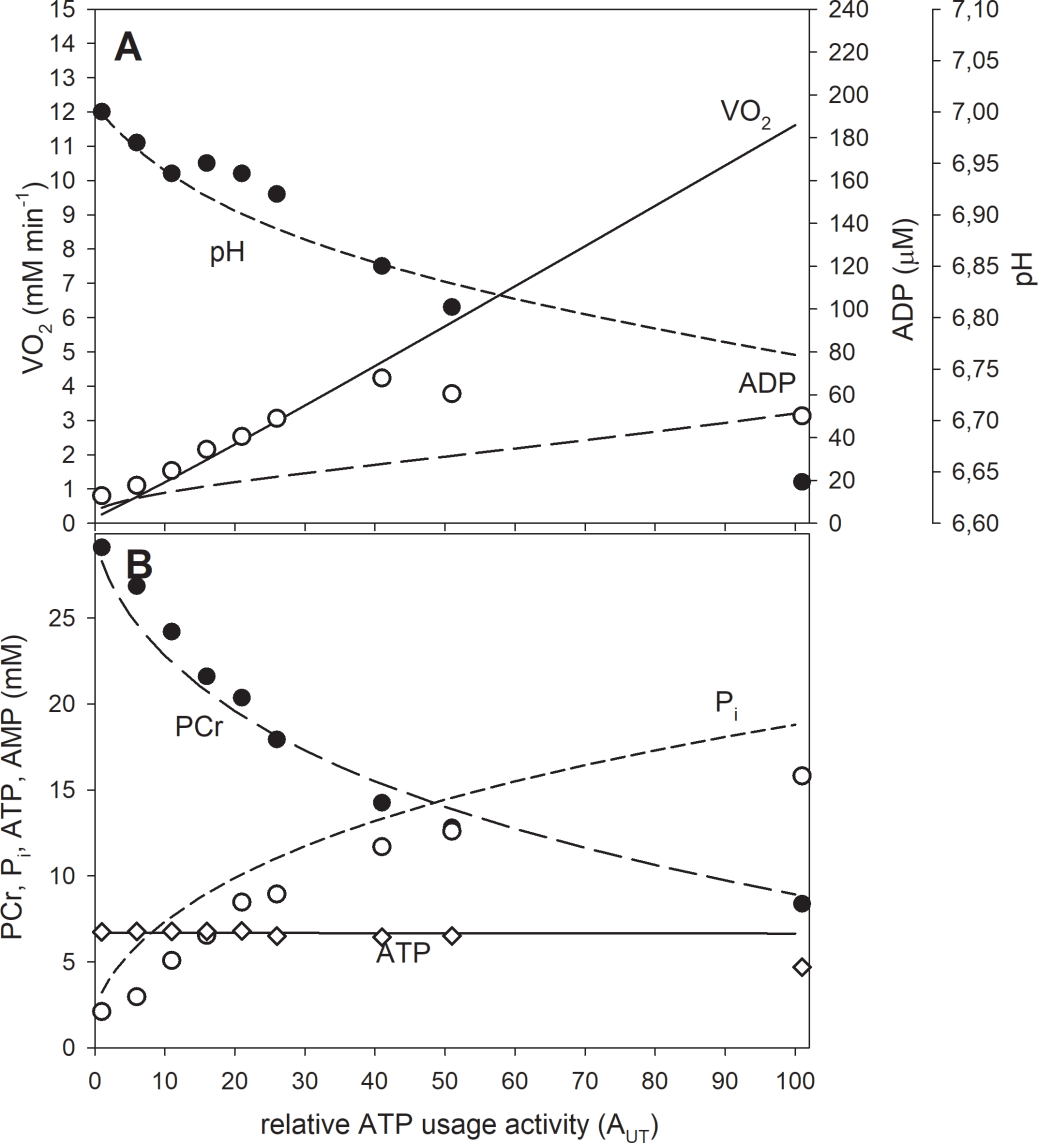
Simulated (lines) and experimental (points) dependence of system variables on relative ATP usage activity A_UT_ for the power-type A_OX_ (ESA, each-step activation intensity)-A_UT_ dependence in the absence of the ‘additional’ ATP usage. A, dependence of $\dot{V}O_{2}$, ADP and pH; B, dependence of PCr, P_i_ and ATP. Re-scaled (see sub-section 2.5) experimental data from [24] are presented (points). The power-type A_OX_-A_UT_ dependence without additional ATP usage is postulated to be present in electrically-stimulated muscle.

**Fig 3:**
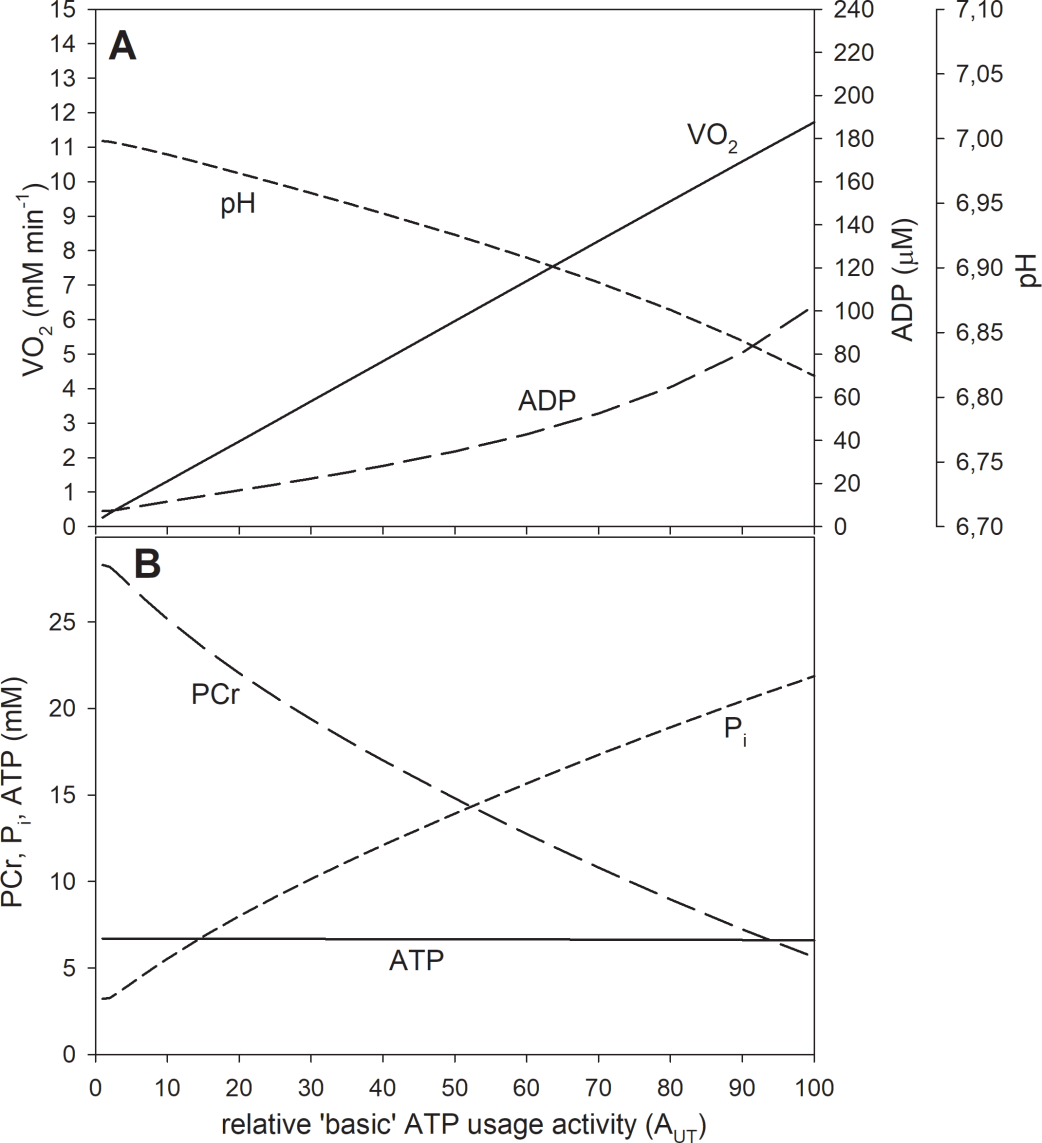
Simulated dependence of system variables on relative ATP usage activity A_UT_ for the saturating-type A_OX_ (ESA, each-step activation intensity)-A_UT_ dependence in the absence of the ‘additional’ ATP usage. A, dependence of $\dot{V}O_{2}$, ADP and pH; B, dependence of PCr, P_i_ and ATP. The saturating-type A_OX_-A_UT_ dependence without additional ATP usage is postulated to be present in voluntary exercise (cortically-stimulated muscle) below critical ATP usage activity (critical power).

**Fig 4:**
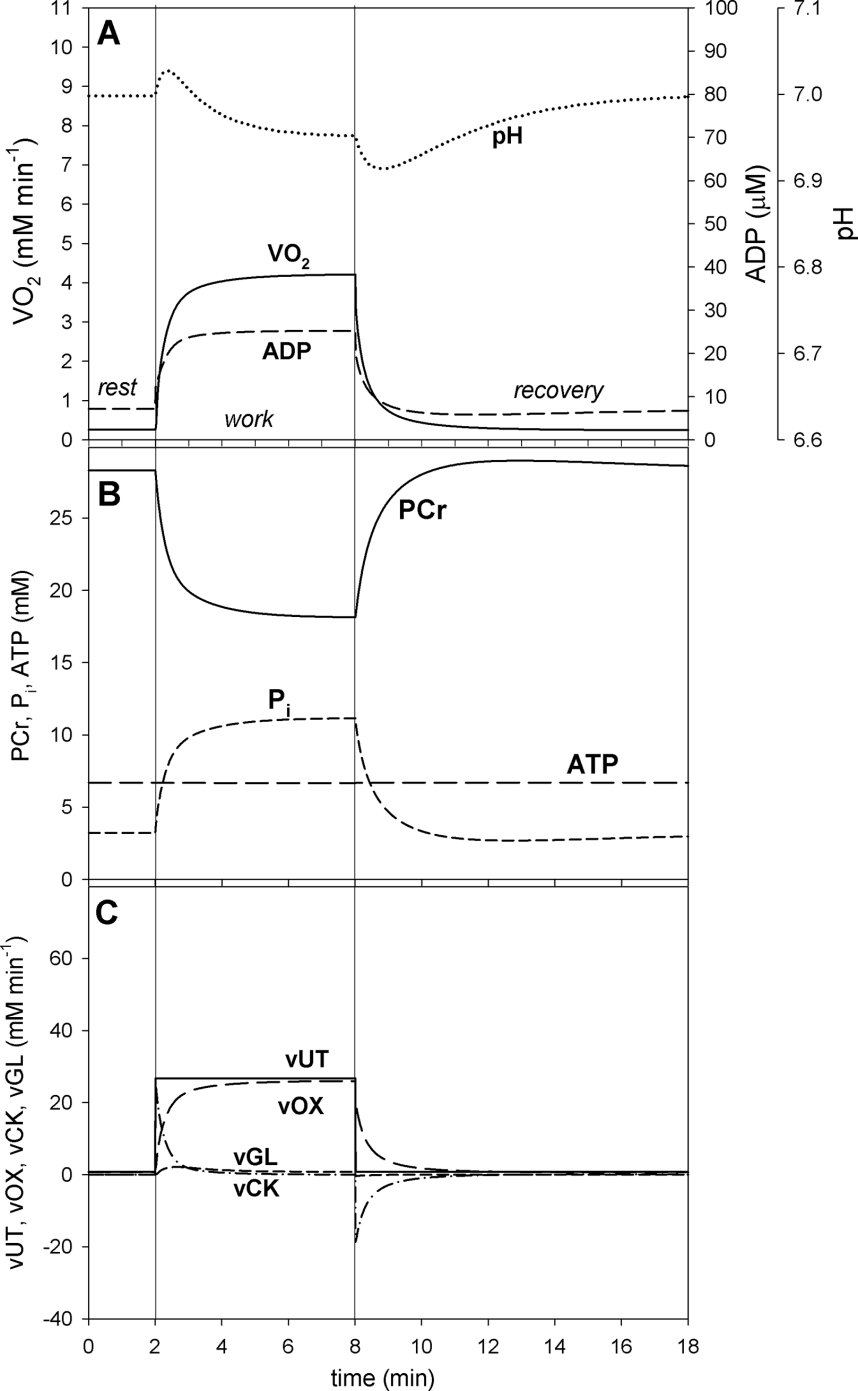
Simulated time courses of system variables during transition from rest to moderate muscle work (relative ATP usage activity A_UT_ = 35) to recovery for the saturating-type A_OX_ (ESA, each-step activation intensity)-A_UT_ (relative ATP usage activity) dependence. A, dependence of $\dot{V}O_{2}$, ADP and pH; B, dependence of PCr, P_i_ and ATP; C, dependence of ATP usage (vUT) as well as of ATP supply by OXPHOS (+ aerobic glycolysis) (vOX), creatine kinase (vCK) and anaerobic glycolysis (vGL). The saturating-type A_OX_-A_UT_ dependence without additional ATP usage is postulated to be present in voluntary exercise below critical ATP usage activity (critical power).

**Fig 5:**
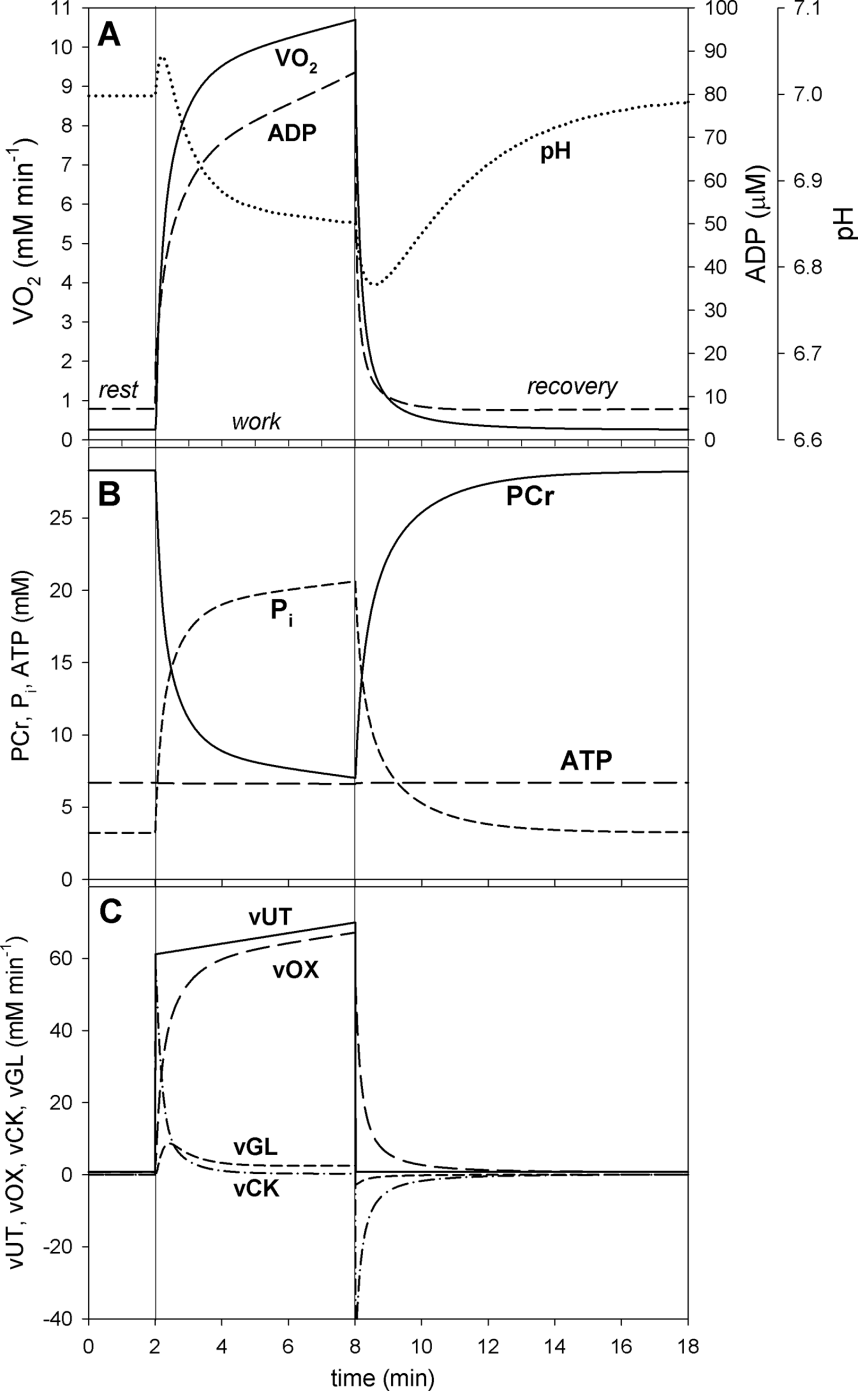
Simulated time courses of system variables during transition from rest to heavy/severe muscle work (relative ATP usage activity A_UT_ = 80) to recovery for the saturating-type A_OX_ (ESA, each-step activation intensity)-A_UT_ (relative ATP usage activity) dependence in the presence of the ‘additional’ ATP usage. A, dependence of $\dot{V}O_{2}$, ADP and pH; B, dependence of PCr, P_i_ and ATP; C, dependence of ATP usage (vUT) as well as of ATP supply by OXPHOS (+ aerobic glycolysis) (vOX), creatine kinase (vCK) and anaerobic glycolysis (vGL). The saturating-type A_OX_-A_UT_ dependence with additional ATP usage is postulated to be present in voluntary exercise above critical ATP usage activity (critical power).

**Fig 6:**
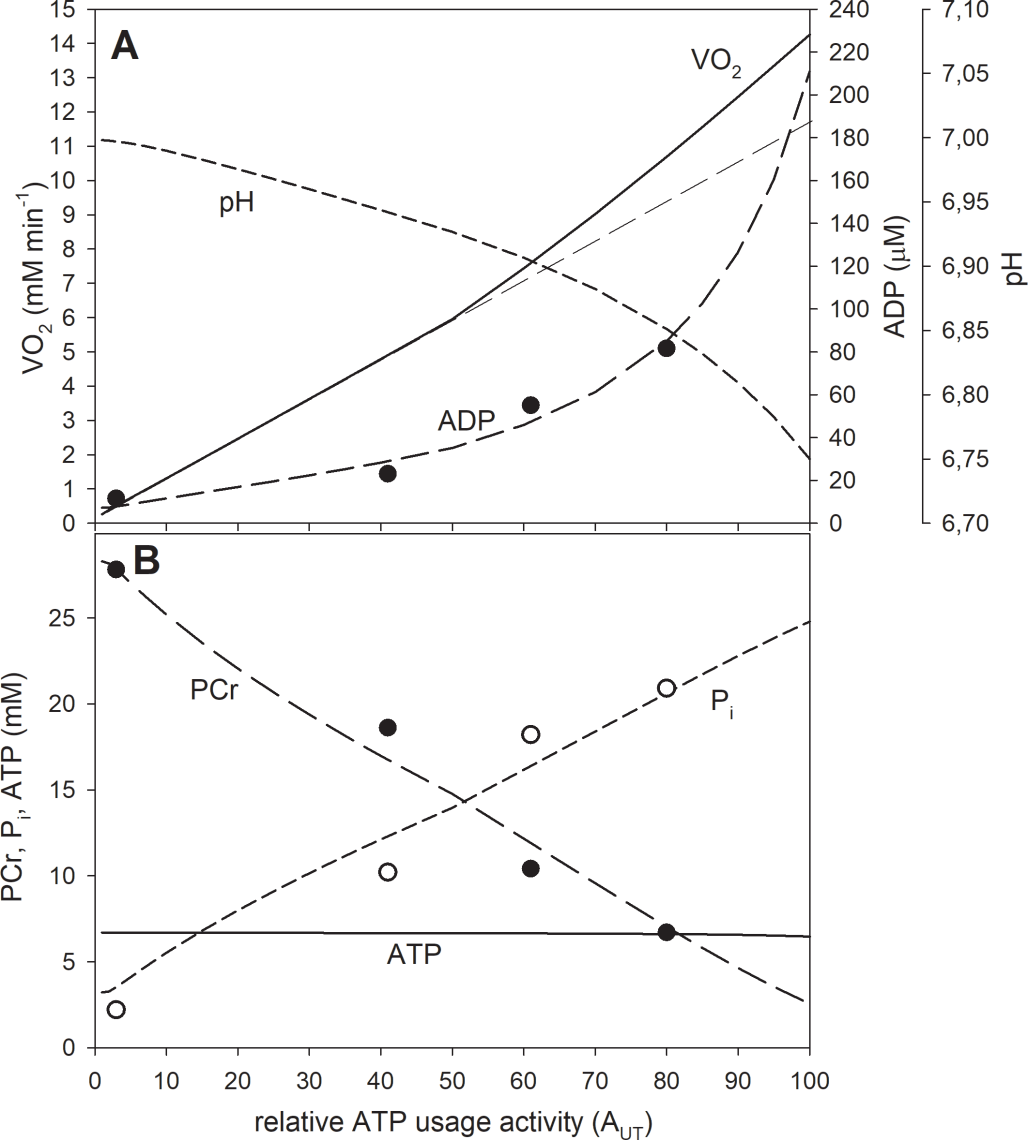
Simulated (lines) and experimental (points) dependence of system variables on relative ATP usage activity A_UT_ for the saturating-type A_OX_ (ESA, each-step activation intensity)-A_UT_ dependence in the presence of the ‘additional’ ATP usage above the critical ATP usage activity. A, dependence of $\dot{V}O_{2}$, ADP and pH; B, dependence of PCr, P_i_ and ATP. Re-scaled (see sub-section 2.5) experimental data for medial gastrocnemius from [14] are presented. The saturating-type A_OX_-A_UT_ dependence with additional ATP usage is postulated to be present in voluntary exercise above critical ATP usage activity (critical power).

In the simulations presented in Figs 2–4 the ‘additional’ ATP usage was absent. Therefore, a steady-state could be reached (or at least approached after 6 min of exercise—see Fig 4). In the simulations presented in Figs 5 and 6 the ‘additional’ ATP usage present above A_UTcrit_, underlying the slow component of the $\dot{V}O_{2}$ on-kinetics, was present. As a result, no steady-state could be reached. In all simulations, the muscle work lasted 6 min after the onset of exercise. The simulations concerning the dependence of selected system variables on the relative ATP usage activity, presented in Figs 2, 3 and 6, were terminated in the 6^th^ min of exercise and the variable values were recorded. In subsequent simulations the relative ATP usage activity A_UT_ was increased gradually from 1 (rest) to 100 in each case (for the power-type A_OX_-A_UT_ dependence and for the saturating-type A_OX_-A_UT_ dependence without and with the ‘additional’ ATP usage). Time courses of system variables during rest-to-work-to-recovery transition were simulated for moderate exercise (A_UT_ = 35) (Fig 4) and heavy/severe exercise (A_UT_ = 80) (Fig 5). Muscle work was initiated in the 2^nd^ min of simulation and terminated after 6 min, in the 8^th^ min of simulation.

The power-type A_OX_ (ESA, each-step activation intensity)-A_UT_ (relative ATP usage activity) dependence was used in the simulations presented in Fig 2, while the saturating-type A_OX_-A_UT_ dependence was used in the simulations presented in Figs 3–6. The ‘additional’ ATP usage was present in the simulations shown in Figs 5 and 6.

The third-order inhibition of glycolysis by H^+^ ions, introduced recently [1], was used in the present study.

The oxygen concentration O_2_ = 30 εM was assumed in all simulations.

**Fig 7:**
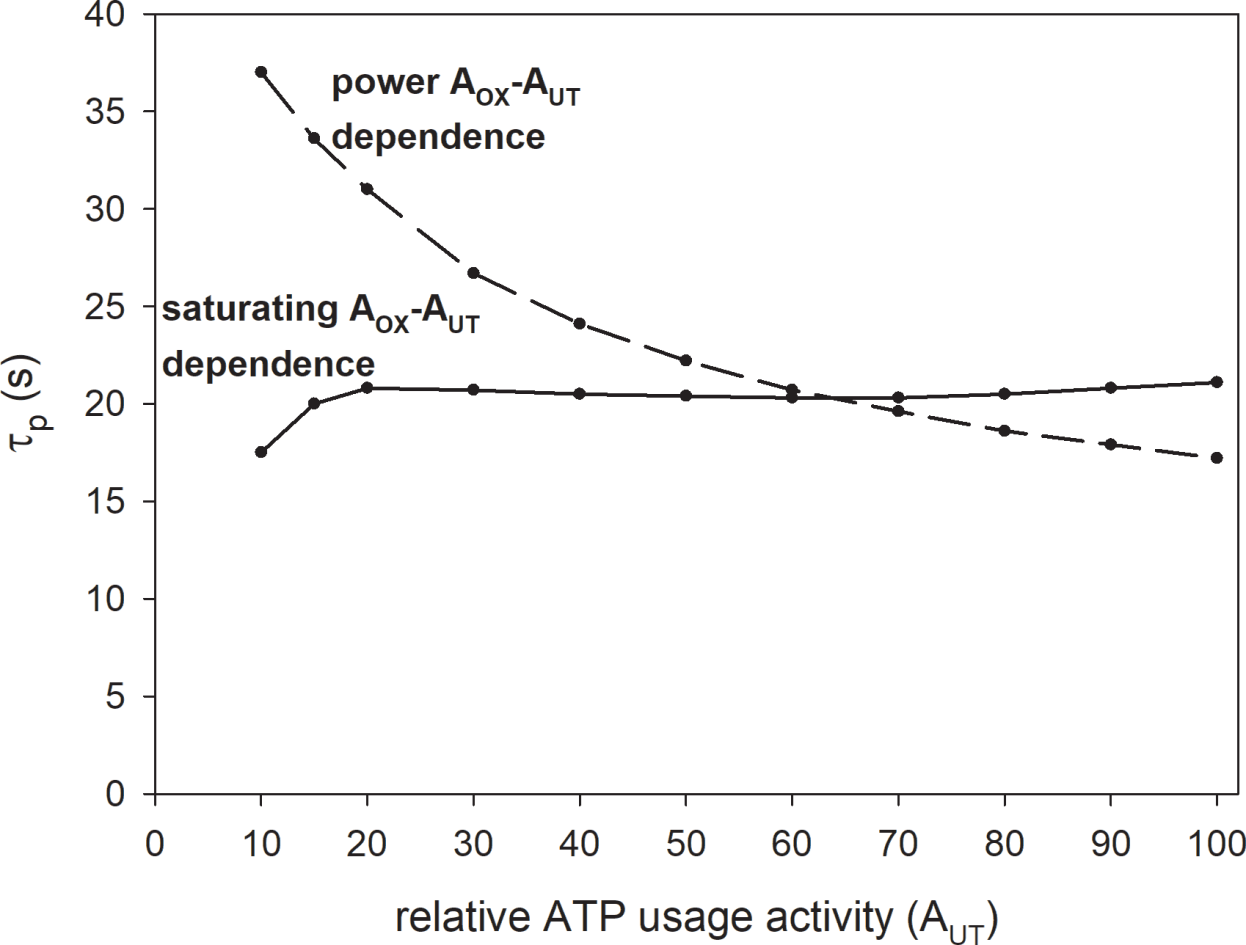
Simulated relationship of the characteristic transition time τ_p_ of the principal phase of the muscle $\dot{V}O_{2}$ on-kinetics on relative ATP usage activity (A_UT_) for the power-type and saturating-type A_OX_ (ESA, each-step activation intensity)-A_UT_ dependencies. The relative activation of OXPHOS during rest-to-work transition A_OX_ was increased as a function of A_UT_ according to Eq 1 for power-type dependence and to Eq 2 for saturating-type dependence. The power-type A_OX_-A_UT_ dependence without ‘additional’ ATP usage is postulated to be present in electrically-stimulated muscle, while the saturating-type A_OX_-A_UT_ dependence with ‘additional’ ATP usage is postulated to be present in voluntary exercise (cortically-stimulated muscle).

τ_p_ of the $\dot{V}O_{2}$ on-kinetics (see Fig 7) was determined for moderate work intensity.

The $\dot{V}O_{2}$ on-kinetics is very different in all-out exercise than in constant-power exercise [23]. However, power output declines very significantly during all-out exercise, while only constant-power exercise is analyzed in the present study.

### Re-scaling of experimental data

In order to make a direct comparison of computer simulations, especially of relative changes in system variable values, with experimental data, some of the experimental data had to be re-scaled, as the system variables were expressed there in different units than that applied in the computer model used. Additionally, while the computer model used is devoted quantitatively to simulate the voluntary whole-body exercise (e.g., cycling) or bipedal knee-extension exercise (two quadricepses involved) in mean humans, the experimental data, with which computer simulations were directly compared, concern rat skeletal muscle stimulated electrically [24] or human calf muscles during voluntary exercise in well-trained Sherpas [14].

**Fig 8:**
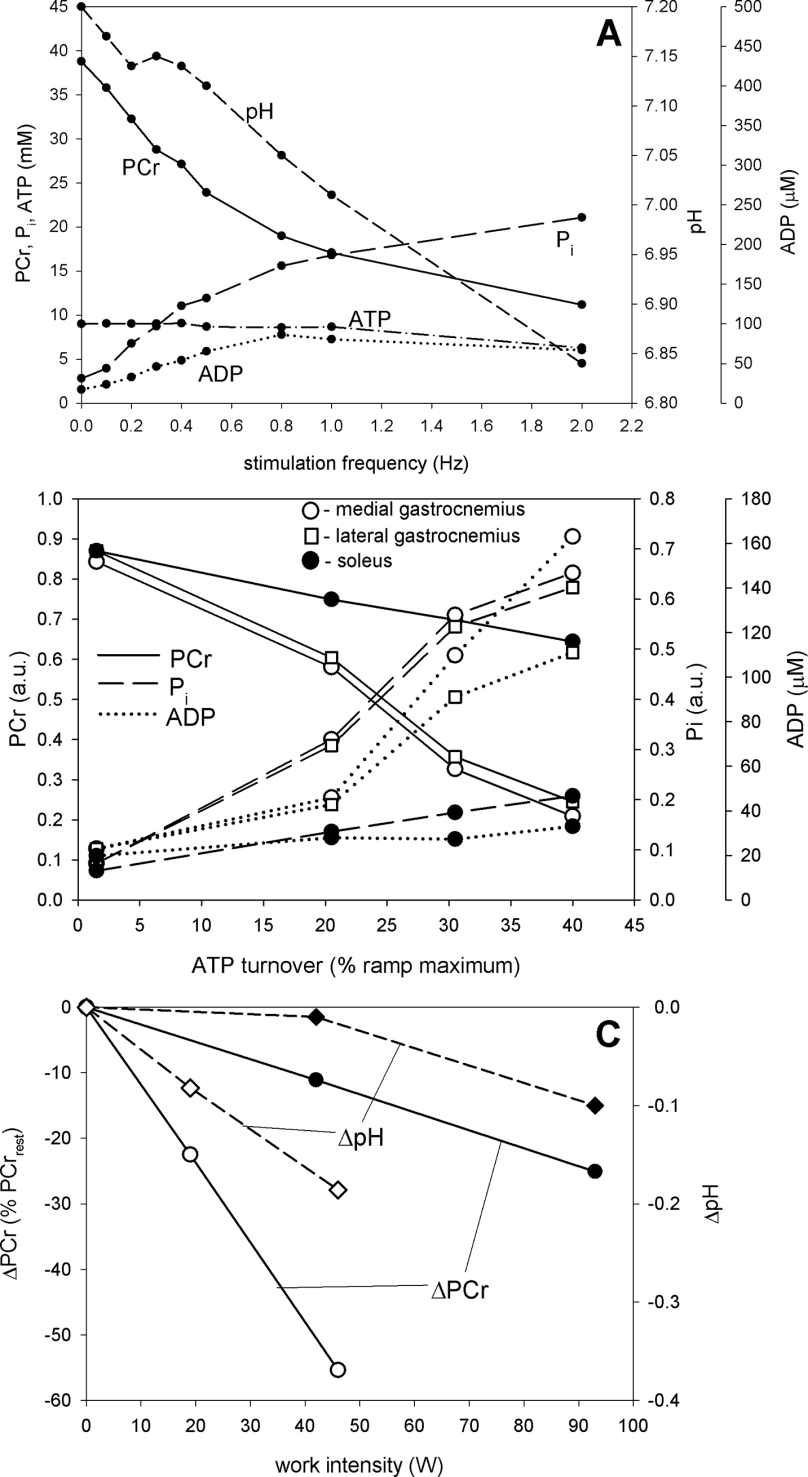
Experimental dependence of skeletal muscle bioenergetic system variables on parameters / variables related to ATP usage activity. A. Original (not re-scaled) dependence of PCr, P_i_, ADP, ATP, pH after 8–12 min of stimulation on electrical stimulation frequency in rat skeletal muscle (Table I and II in [24]). B. Original (not re-scaled) dependence of PCr, P_i_ and ADP after 4 min of exercise on ATP turnover rate (% of maximal) in human calf muscle during voluntary constant-power exercise (pedal pressing) (extracted from Fig 6 in [14]). C. Dependence of the decrease in PCr and pH (in relation to rest) after 6 min of exercise on work intensity in human quadriceps muscles during voluntary constant-power exercise (bilateral knee extension) (closed symbols, [2]; open symbols, [35]).

The data from [24] concern the dependence of PCr, P_i_, ATP and ADP concentrations and pH on muscle electrical stimulation frequency (Hz). Metabolite concentrations are expressed for cellular water and the measured resting pH equals 7.2. On the other hand, metabolite concentrations within the model are expresses for cell volume, the resting pH equals 7.0 and muscle work is expressed as the relative ATP usage activity A_UT_ (unitless). Therefore, the following re-calculations were made: Met_sim_ (mM for cell volume) = Met_exp_ (mM for cellular water) / 1.33 (assuming that water occupies about 75% of the myocyte volume). Met_sim_ is simulated metabolite Met concentration and Met_exp_ is experimental metabolite Met concentration. It was assumed, in order to fit best the simulations to experimental data, that A_UT_ = 1 corresponds to electrical stimulation frequency f_S_ = 0 Hz (rest), while A_UT_ = 101 corresponds to electrical stimulation frequency f_S_ = 2 Hz (intense work), and therefore A_UT_ (unitless) = 50 * f_S_ (Hz) + 1. Re-scaled experimental data from [21] are presented in Fig 2 together with computer simulations, while original data—in Fig 8A.

The data from [14] concern the dependence of PCr, P_i_ and ADP concentrations on relative ATP turnover (% of maximum) in different calf muscles: soleus, lateral gastrocnemius and medial gastrocnemius. PCr and P_i_ concentrations were expressed in arbitrary units. Again, in order to make at least a comparison of simulated and experimental (for medial gastrocnemius) relative changes in particular metabolite concentrations with work intensity, some experimental data re-scaling was necessary. It was assumed that ATP turnover equal to 40% of maximum corresponds to A_UT_ = 80, and therefore A_UT_ (unitless) = 2 * ATP turnover (% of maximum). PCr and P_i_ concentrations were rescaled from arbitrary units (a.u.) to mM using the recalculation factor of 32: Met_sim_ (mM) = Met_exp_ (a.u.) * 32. As calculated absolute ADP levels (they differ significantly between different experiments) were generally higher in the discussed study than that predicted in computer simulations, in order to directly compare the relative changes in ADP the experimental values were reduced by a factor of two. Re-scaled experimental data from [14] for medial gastrocnemius are presented in Fig 6 together with computer simulations, while original data for different calf muscles—in Fig 8B.

### Theoretical results

In the absence of the ‘additional’ ATP usage the relationship between $\dot{V}O_{2}$ and ATP usage activity (A_UT_) is linear regardless the A_OX_ (ESA, each-step activation intensity)-A_UT_ dependence, as one can see in Figs 2 and 3. However, the latter dependence affects significantly the relationship between metabolite concentrations (and pH) and A_UT_. An active (‘working’) steady-state was reached in these simulations– $\dot{V}O_{2}$, metabolite concentrations and pH stabilized during muscle work on constant levels–this can be observed in Fig 4.

The power-type A_OX_ (ESA, each-step activation intensity)-A_UT_ (relative ATP usage activity) dependence causes that PCr, P_i_, ADP and pH change significantly in relation to rest (ADP and P_i_ increase, PCr and pH decrease) already at low A_UT_ values. When A_UT_ increases further towards high values, these changes (except the increase in ADP) slow down–the relationship between PCr, P_i_ and pH, and A_UT_ becomes more flat. Generally, the relationship between PCr and P_i_ concentrations and pH, and the (relative) ATP usage activity is essentially non-linear, especially at low A_UT_ values. On the other hand, ADP increases near-linearly with A_UT_. ATP remains essentially constant. This is demonstrated in Fig 2.

In the case of the saturating-type A_OX_ (ESA, each-step activation intensity)-A_UT_ (relative ATP usage activity) dependence in the absence of the ‘additional’ ATP usage the relationship between PCr, P_i_ and pH, and A_UT_ becomes more linear. These system variables change less in relation to rest at low A_UT_ values, but more at high A_UT_ values, than in the case of the power-type A_OX_-A_UT_ dependence. On the other hand, ADP increases significantly at high A_UT_ values, and the ADP-A_UT_ relationship becomes essentially non-linear. This is presented in Fig 3.

Finally, the ‘additional’ ATP usage, appearing above the critical ATP usage activity, was taken into account in simulations for the saturating-type A_OX_ (ESA, each-step activation intensity)-A_UT_ (relative ATP usage activity) dependence. Of course, in this kind of simulations, an active (‘working’) steady-state cannot be achieved above the critical ATP usage activity (related to critical power, see Discussion), although it is still reached below it. For this reason, in the 6^th^ min of muscle work the values of system variables were recorded, the work was terminated and muscle passed to the recovery phase.

The simulated time courses of selected system variables during rest-to-work-to-recovery transition for moderate work intensity / relative ATP usage activity A_UT_ = 35 (below the critical ATP usage activity A_UTcrit_ = 50) and for high work intensity / relative ATP usage activity A_UT_ = 80 (above the critical ATP usage activity A_UTcrit_ = 50) are demonstrated in Fig 4 and Fig 5, respectively.

It can be seen that at moderate relative ATP usage activity A_UT_ the changes in $\dot{V}O_{2}$ and metabolite levels in time during rest-to-work transition are also moderate. $\dot{V}O_{2}$ increases about 16 times in relation to rest, ADP increases 3.5 times, P_i_ increases 3.5 times, PCr decreases to 66% of the resting value and pH drops slightly by 0.05 pH units (Fig 4A and 4B). A steady-state is reached (or at least approached during the 6 min of exercise), as $\dot{V}O_{2}$, metabolite concentrations and pH stabilize on constant levels. During recovery variable values return to resting values. A pH on-overshoot and off-undershoot, related to H^+^ consumption / production by creatine kinase, can be observed. The total ATP usage activity is constant during muscle work. While during the first < 0.5 min of muscle work a significant fraction of ATP is supplied by creatine-kinase catalyzed reaction, during the rest of muscle work most ATP is produced by OXPHOS, with a very small contribution of anaerobic glycolysis (Fig 4C). During muscle recovery, ATP for PCr re-synthesis is supplied exclusively by OXPHOS.

At high relative ATP usage activity A_UT_ and in the presence of the ‘additional’ ATP usage, changes in time in $\dot{V}O_{2}$ and metabolite levels during rest-to-work transition are much greater than at moderate A_UT_ and they continue to proceed during work. After 6 minutes of muscle work $\dot{V}O_{2}$ increases 41 times in relation to rest, ADP increases 11.6 times, P_i_ increases 6.4 times, PCr decreases to 24% of the resting value and pH drops by 0.17 pH units (Fig 5A and 5B). A steady-state is not reached, as $\dot{V}O_{2}$ increases (the slow component of the $\dot{V}O_{2}$ on-kinetics appears) and metabolite concentrations and pH change continuously during work. During recovery the variable values return to resting values. A pH on-overshoot and off-undershoot can be observed related to H^+^ consumption / production by creatine kinase. The total ATP usage activity (normal + ‘additional’ ATP usage activity) increases gradually during muscle work (due to an increase in the ‘additional’ ATP usage activity). Creatine kinase is the main ATP producer at the onset of muscle work, but ATP supply is quickly taken over by OXPHOS and, to a much smaller extent, by anaerobic glycolysis (Fig 5C). ATP synthesis by OXPHOS during exercise (and, consequently, $\dot{V}O_{2}$) increases continuously (stimulated by the increase in ADP and P_i_) in order to match the elevated total ATP usage (the ‘additional’ ATP usage increasing in time). During muscle recovery, ATP for PCr re-synthesis is supplied exclusively by OXPHOS.

The presence of the ‘additional’ ATP usage affects significantly the dependence of system variables on the relative ATP usage activity A_UT_ for the saturating-type A_OX_ (ESA, each-step activation intensity)-A_UT_ (relative ATP usage activity) dependence. This is demonstrated in Fig 6. First of all, the relationship between $\dot{V}O_{2}$ (determined in the 6^th^ min of exercise) and A_UT_ becomes non-linear. The increase of $\dot{V}O_{2}$ with A_UT_ accelerates above the ‘critical’ ATP usage activity–the $\dot{V}O_{2}$-A_UT_ relationship becomes steeper and a characteristic ‘change point’ [20,25] appears. This is related to the presence of the slow component of the $\dot{V}O_{2}$ on-kinetics resulting from the appearance of the ‘additional’ ATP usage (the causal relation between the slow component and the $\dot{V}O_{2}$-power output nonlinearity in step-incremental exercise was first postulated explicitly by Zoladz and co-workers [20]). PCr and P_i_ concentrations change more in relation to rest at high ATP usage activities, when compared with the simulations for the saturating-type A_OX_ (ESA, each-step activation intensity)-A_UT_ (relative ATP usage activity) dependence without the ‘additional’ ATP usage (Fig 3). This results in even more linear PCr-A_UT_ and P_i_-A_UT_ dependencies. On the other hand, ADP increases very significantly at high A_UT_ values and the ADP-A_UT_ relationship becomes strongly non-linear. The ‘additional’ ATP usage also accelerates the decrease in pH with A_UT_ above the ‘critical’ ATP usage activity–the pH-A_UT_ relationship becomes progressively steeper at high A_UT_ values.

The simulated relationship between system variables and the relative ATP usage activity A_UT_ for the power-type A_OX_ (ESA intensity)-A_UT_ dependence in the presence of the ‘additional’ ATP usage was of course identical as that in the absence of the ‘additional’ ATP usage for A_UT_ (relative ATP usage activity) < A_UTcrit_ (critical ATP usage activity) (not shown) (compare Fig 2). For A_UT_ > A_UTcrit_ a greater decrease in PCr and increase in P_i_ and ADP in the former than in the latter case was predicted. The non-linearity in the $\dot{V}O_{2}$-A_UT_ relationship appeared. Generally, the PCr- and P_i_- A_UT_ dependences remained strongly non-linear, the ADP-A_UT_ relationship become moderately ‘bent upward’, while pH decrease slightly accelerated at higher A_UT_ values (not shown).

The simulated dependence of the characteristic transition time of the principal phase of the muscle $\dot{V}O_{2}$ on-kinetics τ_p_ on the relative ATP usage activity A_UT_ for the power-type and saturating-type A_OX_ (ESA intensity)-A_UT_ dependence is demonstrated in Fig 7. One can see that in the first case τ_p_ decreases with A_UT_, while in the second case it remains essentially constant, apart from the lowest A_UT_ values, where it moderately increases with A_UT_. Generally, the simulated τ_p_ values are rather low (but still well within the values reported for human subjects), because a relatively high ESA intensity was used in these simulations and τ_p_ depends significantly on ESA intensity [26]. When a lower ESA intensity was used in computer simulations (e. g., A_OXmax_ = 3–4.5), longer τ_p_s were obtained (28–40 s). In extreme cases, the simulated τ_p_ can be lower than 10 s (for A_OXmax_ > ~ 12) or higher than 50 s (for A_OXmax_ = 2) (see also [7,26,27]). Therefore, the ESA-dependent range of τ_p_ covers the entire range of τ_p_ encountered in humans, from very well-trained athletes to elderly people and patients with numerous diseases (see e.g., [28]). However, τ_p_ depends also on the resting (without ESA) OXPHOS activity / mitochondria content [26].

## Discussion

The present theoretical study demonstrates that the power-type A_OX_ (ESA, each-step activation intensity)-A_UT_ (relative ATP usage activity) in the absence of the ‘additional’ ATP usage predicts significantly different kinetic behavior of the bioenergetic system in skeletal muscle than the saturating-type A_OX_-A_UT_ dependence in the presence of the ‘additional’ ATP usage. In the former case, $\dot{V}O_{2}$ increases linearly with A_UT_, the dependence of PCr, cytosolic P_i_ and pH on A_UT_ is strongly non-linear (large changes at low A_UT_ values, smaller changes at higher A_UT_ values), while ADP increases near-linearly with A_UT_. On the other hand, in the latter case, the $\dot{V}O_{2}$-A_UT_ depends is significantly non-linear (it bends upward above the critical ATP usage activity), PCr decreases and P_i_ increases near-linearly with A_UT_, the ADP-A_UT_ and pH-A_UT_ dependence is strongly non-linear (the increase in ADP and decrease in pH with A_UT_ accelerates at higher A_UT_ values). As it is discussed below, computer simulations using the power-type A_OX_-A_UT_ dependence reproduce well experimental data for electrically-stimulated skeletal muscle, while simulations using the saturating-type A_OX_-A_UT_ dependence in the presence of the additional ATP usage (underlying the slow component of the $\dot{V}O_{2}$ on-kinetics) are able to account satisfactorily for the kinetic behavior of the bioenergetic system in cortically-stimulated skeletal muscle (voluntary exercise in humans). It is argued that the difference between the electrically- and cortically stimulated muscle results from different patterns of various muscle fibers recruitment. Generally, it is concluded that the regulation of OXPHOS is different in electrically- and cortically-stimulated skeletal muscle.

### Power-type vs. saturating-type A_OX_-A_UT_ dependence

It has been shown previously [10,29] that in the absence of ESA huge changes in metabolite (ADP, PCr, P_i_, ATP) levels already at low and moderate work intensities take place. When the relative ATP usage activity A_UT_ reaches the value of about 30, OXPHOS capacity becomes saturated, as an increase in ADP and P_i_ cannot further activate it, muscle $\dot{V}O_{2}$ reaches its maximum at less than 4 mM min^-1^ and the system collapses (PCr and ATP fall to zero).

Computer simulations carried out in the present study show that in the presence of ESA (each-step activation of OXPHOS complexes and NADH supply), but in the absence of the ‘additional’ ATP usage, the $\dot{V}O_{2}$-A_UT_ relationship is linear even at high A_UT_ (relative ATP usage activity) values, as it can be seen in Figs 2 and 3. The power-type A_OX_ (ESA intensity)-A_UT_ dependence generates PCr-A_UT_, P_i_-A_UT_ and pH-A_UT_ relationships that are strongly non-linear: PCr, P_i_ and pH change quickly with the A_UT_ increase at low A_UT_ values, but these changes slow down significantly at higher A_UT_ values. On the other hand, the ADP-A_UT_ relationship is near-linear. This is demonstrated in Fig 2. These simulated system properties result from relatively low OXPHOS stimulation by ESA at low A_UT_ values, but relatively strong OXPHOS stimulation by ESA at high A_UT_ values in the case of the power-type A_OX_-A_UT_ dependence (compare Fig 1).

The kinetic behavior of the system is significantly different for the saturating-type A_OX_ (ESA, each-step activation intensity)-A_UT_ (relative ATP usage activity) dependence in the absence of the ‘additional’ ATP supply (Fig 3). Namely, relatively little changes in PCr, P_i_, pH and ADP with the A_UT_ increase at low A_UT_ values can be observed. Changes in PCr and P_i_ only slightly slow down at high A_UT_ values and the PCr-A_UT_ and P_i_-A_UT_ relationships become much more linear than for the power-type A_OX_-A_UT_ dependence. This is caused by relatively high OXPHOS stimulation by ESA already at low A_UT_ values and by the fact that this stimulation does not increase further at higher A_UT_ values in the case of the saturating-type A_OX_-A_UT_ dependence (compare Fig 1). On the other hand, the decrease in pH with A_UT_ is slightly faster at higher A_UT_ values (Fig 3), unlike for the power-type A_OX_-A_UT_ dependence. This is demonstrated in Fig 3.

### Impact of ‘additional’ ATP usage

The inclusion of the ‘additional’ ATP usage [1] above the critical ATP usage activity (A_UTcrit_) in the case of the saturating-type A_OX_ -A_UT_ dependence causes that the system cannot reach a steady-state for A_UT_ (relative ATP usage activity) > A_UTcrit_ (critical ATP usage activity). This can be seen when one compares simulations of rest-to-work-to-recovery transition for moderate work (A_UT_ = 35 < A_UTcrit_ = 50) (Fig 4) and heavy / severe work (A_UT_ = 80 > A_UTcrit_ = 50) (Fig 5).

The critical relative ATP usage activity A_UTcrit_ is strictly related to critical power, that is the power output above which it is not possible to reach a steady-state [30,31]. As a result, above critical power exercise cannot be continued for a long (potentially unlimited) time [30,31].

In heavy / severe exercise system variables (muscle $\dot{V}O_{2}$, PCr, P_i_, ADP, pH) change significantly immediately after the onset of exercise and then, unlike in moderate exercise, continue to change continuously with slower pace, never reaching a steady state. This is demonstrated in Fig 5 vs. Fig 4. The total ATP usage activity increases gradually during exercise, reflecting the increase in the ‘additional’ ATP usage activity. This leads, through an increase in ADP and P_i_, to a slow continuous increase in ATP supply by OXPHOS in order to match the elevated ATP consumption, and consequently a continuous increase in muscle $\dot{V}O_{2}$. The last phenomenon has been named the ‘slow component’ of the $\dot{V}O_{2}$ on-kinetics [32]. The pulmonary $\dot{V}O_{2}$ slow component is generated principally within the exercising skeletal muscles [32,33].

The presence of the ‘additional’ ATP usage in the system with the saturating-type A_OX_ (ESA, each-step activation intensity)-A_UT_ (relative ATP usage activity) dependence affects significantly the system variables-A_UT_ relationships. PCr-A_UT_ and P_i_-A_UT_ relationships become near-linear, while the ADP-A_UT_ relationship becomes strongly non-linear. The decrease of pH with A_UT_ accelerates significantly above the critical ATP usage activity (related to critical power). This is demonstrated in Fig 6. Finally, the $\dot{V}O_{2}$-A_UT_ relationship becomes essentially non-linear: above the critical ATP usage activity the increase of $\dot{V}O_{2}$ with A_UT_ accelerates and the $\dot{V}O_{2}$-A_UT_ relationship has an increasingly steeper slope. A characteristic ‘change point’ [20,25] appears. This system property is related to the presence of the slow component of the $\dot{V}O_{2}$ on-kinetics resulting from the appearance of the ‘additional’ ATP usage. The causal relation between the slow component and the $\dot{V}O_{2}$-PO nonlinearity was first postulated explicitly for step-incremental exercise (increase by 30 W after each 3 min) by Zoladz and co-workers [20].

### Comparison of computer simulations with experimental data

Selected experimental data concerning the dependence of system variables (PCr, P_i_, ATP, ADP, pH) on some parameter / variable related to the ATP usage activity in different experimental systems are presented in Fig 8. Some of these data, after re-scaling, are also shown in Fig 2 and Fig 6 in order to make direct comparison of these data (especially of relative changes in metabolites and pH) with computer simulations. Preference was given to measurements of metabolite concentrations / pH using ^31^P MRS over chemical determination in muscle biopsies and to sufficiently wide range of electrical stimulation frequency or work intensity values (assumed to be proportional in a given type of exercise to the ATP usage activity).

In rat skeletal muscle stimulated electrically [24] the fall in PCr and rise in P_i_ with the stimulation frequency (after 8–12 min of exercise) is relatively quick at low stimulation frequencies, but slows down at higher stimulation frequencies. This is demonstrated in Fig 8A. Such a kinetic behavior is decidedly similar to the pronounced non-linearity of the PCr-A_UT_ and P_i_-A_UT_ relationship simulated for the power-type A_OX_ (ESA, each-step activation intensity)-A_UT_ (relative ATP usage activity) dependence in the absence of the ‘additional’ ATP usage. This is directly demonstrated in Fig 2, where computer simulations are compared with re-scaled experimental data (see sub-section 2.5) presented in Fig 8A. The experimental and theoretical PCr-A_UT_ and P_i_-A_UT_ dependencies are decidedly similar. Also the experimental and theoretical ATP-A_UT_ dependencies are similar, although experimental ATP somewhat decreases at highest stimulation frequencies, most probably due to AMP deamination. This process was not involved in the present study, but its impact on the system was analyzed previously [34] (see below for discussion). The simulated pH-A_UT_ dependence agrees well with the measured pH decrease with stimulation frequency, apart from one experimental point for the highest stimulation frequency, in which the measured pH falls in relation to rest significantly more than the simulated one. The experimental value of ADP calculated from CK equilibrium increases linearly with stimulation frequency for low and moderate frequencies, like in computer simulations. The absolute ADP concentration is higher in the former case, than in the latter case, but it could not be directly measured in the experiment, but was calculated from the creatine kinase equilibrium involving cytosolic pH. As the absolute resting pH value in the computer model equals 7.0, while in the experiment: 7.2, the differences in the absolute ADP concentration result mostly from this fact. At higher stimulation frequencies ADP slightly falls down in the experiment, while it slightly increases in the simulations presented in Fig 2. However, a decrease of ADP with time (after about 2 min of exercise) at high ATP usage activities was simulated in the presence of intensive AMP deamination [34]. This fact can potentially explain the discussed apparent discrepancy. Generally, the kinetic behavior of the system in electrically-stimulated rat skeletal muscle is well reproduced by computer simulations involving the power-type A_OX_ (ESA, each-step activation intensity)-A_UT_ (relative ATP usage activity) dependence in the absence of the ‘additional’ ATP usage (Fig 2). Nevertheless, it must be stressed that during experimental data re-scaling (recalculation) it was assumed that the ATP usage activity is linearly related to the stimulation frequency. This may be not true at high stimulation frequencies, where electrically-stimulated muscle approaches tetani and ATP usage does not further increase with stimulation frequency.

**Fig 9:**
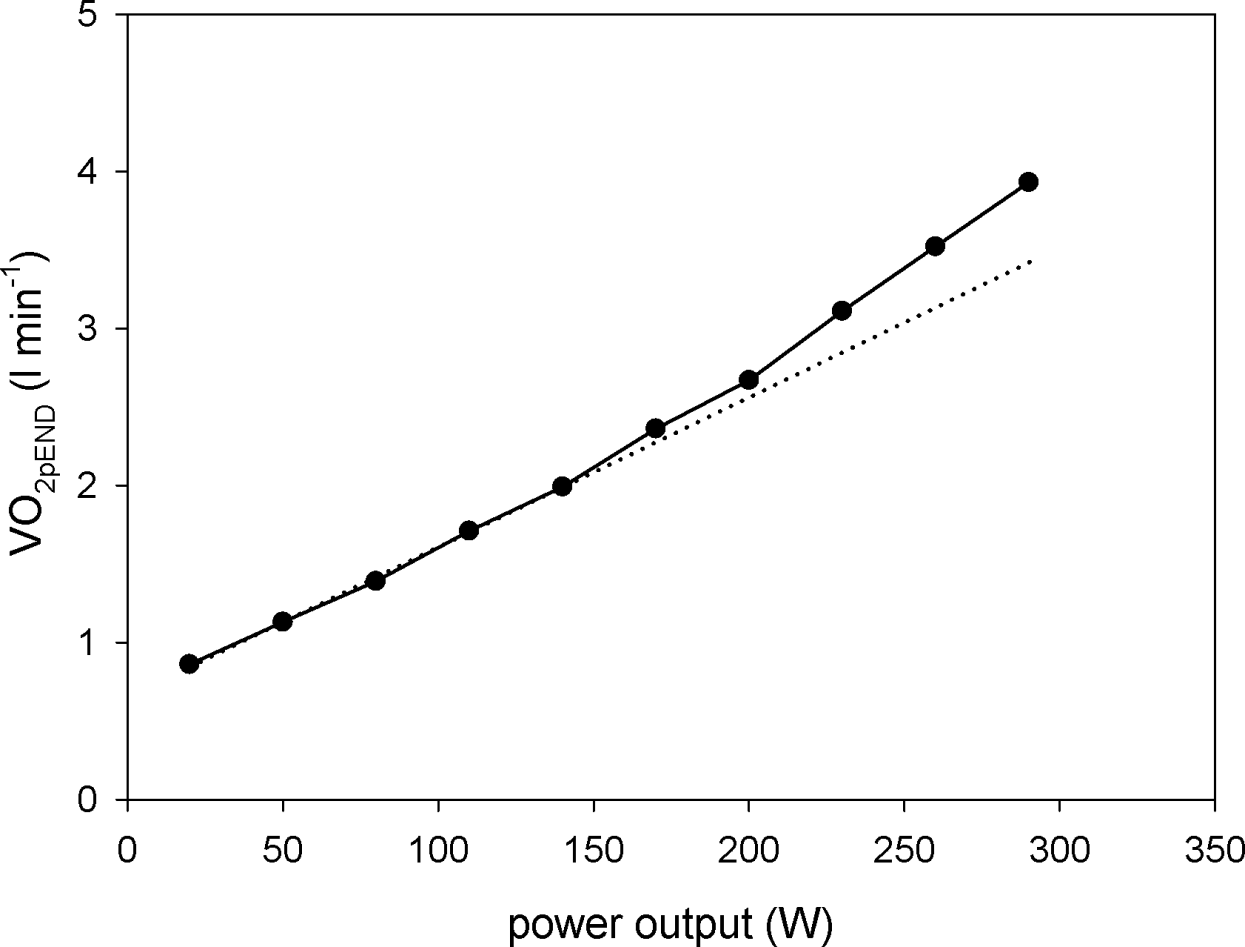
Experimental $\dot{V}O_{2p}$-power output dependence. Experimental dependence of $\dot{V}O_{2\mathrm{pEND}}$ (oxygen consumption at the end of subsequent steps) on the power output (PO) in subsequent steps in step-incremental exercise (increase in PO by 60 W after each 6 or 8 min in two overlapping protocols ‘shifted in phase’ by 30 W, with a baseline of 20 W and 50 W) extracted from Table 1 in [19] is presented.

In human calf muscles in voluntary pedal-pressing exercise (cortical muscle stimulation) [14] the dependence of relative PCr and P_i_ levels on ATP turnover (usage) is near-linear. On the other hand, the increase in ADP with ATP turnover is strongly nonlinear, as it significantly accelerates at high ATP turnover rates. This is shown in Fig 9B. This kind of the kinetic behavior of the system is decidedly more similar to the simulated dependence of system variables on A_UT_ (relative ATP usage activity) for the saturating-type A_OX_ (ESA, each-step activation intensity)-A_UT_ dependence without the ‘additional’ ATP usage (Fig 3) than for the power-type A_OX_-A_UT_ dependence (Fig 2). However, the pattern of the relationship between PCr, P_i_ and ADP, and ATP turnover is even more similar to the model predictions for the saturating-type of the A_OX_-A_UT_ dependence in the presence of the ‘additional’ ATP usage, shown in Fig 6. In this figure, re-scaled data for medial gastrocnemius from [14] are directly compared with computer simulations for the saturating-type A_OX_-A_UT_ dependence in the presence of the ‘additional’ ATP usage. The experimental and theoretical PCr-A_UT_, P_i_-A_UT_ and ADP-A_UT_ dependencies are decidedly similar. The absolute (not re-scaled) ADP concentration is higher in the experimental data than in computer simulations. However, the experimental ADP level was not measured directly, but calculated from the creatine kinase equilibrium involving pH that is not shown in [14]. Generally, the saturating-type A_OX_ (ESA, each-step activation intensity)-A_UT_ (relative ATP usage activity) dependence in the presence of the ‘additional’ ATP usage seems to be able to account well for the behavior of the bioenergetic system in cortically-stimulated skeletal muscle during voluntary exercise in humans.

In human quadriceps muscle in voluntary bipedal knee extension exercise (cortical muscle stimulation) [2,35] the dependence of the decrease in PCr (ΔPCr) after 6 min of moderate and heavy / severe exercise in relation to rest on the work intensity (proportional to A_UT_) is also linear. This is shown in Fig 8C. The ΔpH-work intensity relationship is linear in [35], while the pH decrease with work intensity accelerates at high work intensities in [2]. Generally, these data seem to agree much better with computer simulations for the saturating-type A_OX_ (ESA, each-step activation intensity)-A_UT_ (relative ATP usage activity) dependence (Fig 3), especially in the presence of the ‘additional’ ATP usage (Fig 6), than with simulations for the power-type A_OX_-A_UT_ dependence (Fig 2).

The simulated muscle $\dot{V}O_{2}$-A_UT_ (relative ATP usage activity) relationship (for the 6^th^ min after the onset of exercise) for the saturating-type A_OX_ (ESA, each-step activation intensity)-A_UT_ (relative ATP usage activity) dependence in the presence of the ‘additional’ ATP usage is essentially non-linear. Such a non-linearity in the pulmonary $\dot{V}O_{2}$-PO (A_UT_) relationship was first postulated explicitly and explained for step-incremental exercise (30 W /30 min) by Zoladz and co-workers [20,25]. Fig 9 presents a similar pulmonary $\dot{V}O_{2}$-PO (A_UT_) dependence extracted from a recent study by Keir and co-workers [19].

Generally, it seems evident that the power-type A_OX_ (ESA, each-step activation intensity)-A_UT_ (relative ATP usage activity) dependence reproduces well the kinetic system behavior in electrically-stimulated muscle, while the saturating-type A_OX_-A_UT_ dependence in the presence of the ‘additional’ ATP usage can account much better for the kinetic properties of the bioenergetic system in cortically-stimulated muscle during voluntary exercise. Therefore, the regulation of this system, especially of OXPHOS, seems to be significantly different in these two types of muscle stimulation.

### τ_p_—A_UT_ relationship

Under most conditions, the pulmonary $\dot{V}O_{2}$ on-kinetics seems to represent well the muscle $\dot{V}O_{2}$ on-kinetics for constant-power exercise [36,37]. Therefore, both kinetics can be directly compared for this kind of exercise, although some dissociation of the pulmonary and muscle $\dot{V}O_{2}$ on-kinetics, especially at high work intensities, cannot be excluded.

An essentially constant τ_p_ of the principal component (phase II) of the pulmonary $\dot{V}O_{2}$ on-kinetics, independent of PO, was encountered in several studies for voluntary constant-power exercise (cortically-stimulated muscle) in humans [2,5]. This is demonstrated in Table 1. One can see that τ_p_ does not depend, within experimental error, on the work intensity in this type of exercise. This agrees well with the model predictions for the saturating-type A_OX_ (ESA, each-step activation intensity)-A_UT_ (relative ATP usage activity) dependence concerning the τ_p_-A_UT_ relationship shown in Fig 7.

**Table 1:** Experimental values of the characteristic transition time τ_p_ of the $\dot{V}O_{2}$ on-kinetics at different bilateral knee extension exercise intensities for the same group of individuals in each experiment.

reference	[5]	[2]
Work intensity:		
moderate	33 ± 16 s	35 ± 14 s
heavy	32 ± 17 s	39 ± 4 s
very heavy	34 ± 11 s	
severe	34 ± 7 s	

A much wider review [38] (see Table 12.2 therein) of different experimental data for constant-power voluntary exercise in humans demonstrates that in almost half of the cases (experiments) τ_p_ was almost constant or increased little with work intensity. An average increase in 26 experiments was 21%. Generally, the computer simulations for the saturating-type A_OX_ (ESA, each-step activation intensity)-A_UT_ (relative ATP usage activity) dependence in the presence of the ‘additional’ ATP usage agree fairy well with experimental data. Perhaps, the τ_p_-A_UT_ relationship is more flat in well-trained subjects with more oxidative muscles and less flat for sedentary subjects with less oxidative muscles. Additionally, these experimental data concern τ_p_ for pulmonary $\dot{V}O_{2}$, while muscle $\dot{V}O_{2}$ kinetics is simulated using the computer model. Pulmonary and muscle $\dot{V}O_{2}$ kinetics can slightly dissociate, especially at higher work intensities.

On the other hand, τ_p_ decreases with the relative ATP usage activity A_UT_ in oxidative frog muscle fibers stimulated electrically [39]. This fact agrees very well with computer simulations for the power-type A_OX_-A_UT_ dependence in the absence of the ‘additional’ ATP usage shown in Fig 7.

Again, it seems that the regulation of OXPHOS is different in electrically- and cortically-stimulated skeletal muscle during constant-power exercise: in the former the A_OX_ (ESA, each-step activation intensity)-A_UT_ (relative ATP usage activity) dependence is power-type and the ‘additional’ ATP usage is absent, while in the latter the A_OX_-A_UT_ dependence is saturating-type-type and the ‘additional’ ATP usage is present.

A (very) significant increase in τ with PO was observed in experiments concerning voluntary cycling step-incremental exercise [19]. However, in this exercise mode, predominantly glycolytic type II (especially IIx and IIb) muscle fibers, with high τ_p_ values, can be recruited at the onset of subsequent steps at high work intensities, what would explain this phenomenon [40].

### Each-step activation (ESA) mechanism

A general mechanism of parallel activation of ATP usage and ATP supply during skeletal muscle contraction was first postulated by Hochachka and Matheson [41,42]. It is supported by the observation [43] that the first phase of the $\dot{V}O_{2}$ increase after the onset of exercise is ADP-independent, and therefore $\dot{V}O_{2}$ must be quickly elevated by some other mechanism. This mechanism was also confirmed by Wüst and co-workers on the basis of the $\dot{V}O_{2}$-ADP relationship during on-transient in skeletal muscle [44].

Fell and Thomas [45] proposed, in a more general and abstract way, an idea similar to ESA, called ‘multi-site modulation’, in relation to other metabolic pathways, especially glycolysis.

The each-step activation (ESA) mechanism, being a special case of the parallel-activation mechanism, was proposed in relation to OXPHOS in skeletal muscle by Korzeniewski [6]. According to this mechanism, not only ATP usage, but also all OXPHOS complexes (complex I, complex III, complex IV, ATP synthase, ATP/ADP carrier, P_i_ carrier), NADH supply block and glycolysis / glycogenolysis are directly activated by some cytosolic mechanism, together with the stimulation of muscle contraction (actomyosin-ATPase and Ca^2+^-ATPase) by Ca^2+^ ions.

Behnke and co-workers [22] encountered a significant increase in $\dot{V}O_{2}$ immediately after the onset of electrical muscle simulations. This agrees well with computer simulations (Figs 4 and 5), where a very quick increase in $\dot{V}O_{2}$ takes place after muscle stimulation due to each-step activation of OXPHOS complexes (very short characteristic activation time t(ON)_OX_ = 3 s) and ADP increase.

The molecular mechanism of ESA is still not fully known. Activation by matrix Ca^2+^ of three irreversible TCA (tricarboxylic acid) cycle dehydrogenases (pyruvate dehydrogenase, isocitrate dehydrogenase, 2-oxoglutarate dehydrogenase) [46,47] and by cytosolic Ca^2+^ of aralar, an element of the malate / aspartate shuttle (MAS) [48] has been proposed. Glancy and co-workers [49] showed that Ca^2+^ elevated about 2-fold the activity of essentially all OXPHOS complexes in isolated skeletal muscle mitochondria incubated with glutamate / malate. However, it is not certain whether such an activation is sufficient to account for experimental data. A recent theoretical study [1] suggests that OXPHOS complexes are activated directly over 5-fold during rest-to-work transitions in humans (compare Fig 1). In other muscles or experimental conditions this direct activation of OXPHOS seems to be even higher (see [10] for discussion).

### ATP usage activity and work intensity

It can be assumed that, in a given type of exercise (e.g., voluntary cycling, knee-extension or pedal pressing with a constant determined pace), the ‘normal’ ATP usage (without ‘additional’ ATP usage) increases approximately linearly with mechanical work (power output) (both unloaded and loaded). In the simulations carried out in the present study, when the relative ATP usage activity A_UT_ increases from 50 to 100 (by 50 A_UT_ units), muscle $\dot{V}O_{2}$ (in the absence of the ‘additional’ ATP usage) increases from 5.96 to 11.73 mM min^-1^, and thus muscle $\Delta \dot{V}O_{2}$ = 5.77 mM min^-1^ (Fig 3). Assuming that during intense cycling exercise 85% of pulmonary $\dot{V}O_{2}$ is of working muscle origin [33,50] and that there is 10 kg of intensively working muscles in cycling exercise, this gives the pulmonary $\Delta \dot{V}O_{2}$ equal to 1520 mL min^-1^. Assuming that gain ($\Delta \dot{V}O_{2p}$ / ΔW) g = 10 mL min^-1^ W^-1^ in cycling exercise in humans (after subtraction of the slow component of the $\dot{V}O_{2}$ on-kinetics), we obtain the power output difference ΔW = 152 W. Thus, 1 A_UT_ unit corresponds to about 3.04 W (152 W / 50 A_UT_ units) of work intensity (both unloaded and loaded). Therefore, after subtracting 15 W for unloaded work, 100 A_UT_ units in Figs 2, 3 and 6 corresponds to about 289 W of loaded work (power output) and to about 3040 mL min^-1^ of pulmonary $\Delta \dot{V}O_{2}$ during cycling exercise in humans. However, because of the ‘additional’ ATP usage (leading to the slow component of the $\dot{V}O_{2}$ on-kinetics), the actual pulmonary and muscle oxygen consumption is higher. The muscle $\dot{V}O_{2}$ = 14.6 mM min^-1^ at A_UT_ = 100 after 6 min of exercise for the saturating type A_OX_ (ESA, each-step activation intensity)-A_UT_ (relative ATP usage activity) dependence with the additional ATP usage (Fig 6). This is equivalent to the pulmonary $\dot{V}O_{2}$ = ~ 3850 mL min^-1^. Therefore, in these simulations, the value of the ‘additional’ $\dot{V}O_{2}$ for A_UT_ = 100 (after 6 min of exercise), related to the non-linearity in the $\dot{V}O_{2}$-PO relationship, equals 27%.

### Background of power-type and saturating-type A_OX_-A_UT_ dependence

In the author’s opinion, the difference in the A_OX_ (ESA, each-step activation intensity)-A_UT_ (relative ATP usage activity) dependence between voluntary exercise in humans and electrically-stimulated skeletal muscle in rat is related to the kind of muscle stimulation and, consequently, to the muscle fiber recruitment pattern when ATP usage activity increases. In electrically-stimulated muscle, all types of muscle fibers (type I, IIa and IIb/x fibers) are recruited in parallel already at lowest stimulation frequencies (ATP usage activities), and ATP usage activity increases as a result of an increase in stimulation frequency. Therefore, the contribution of more oxidative type I fibers and less oxidative type II fibers to ATP usage is similar at low and high work intensities. On the other hand, in cortically-stimulated muscle, more oxidative (with higher resting OXPHOS activity and each-step activation (ESA) intensity) type I fibers are recruited predominantly at lower work intensities, while less oxidative (with lower resting OXPHOS activity and ESA intensity) type II muscle fibers start to be recruited at higher work intensities. Therefore, ESA intensity increases with work intensity at higher work intensities in electrically-stimulated muscle, as type I fibers are stimulated more and more with an increase in ATP usage activity (stimulation frequency). On the other hand, most (all?) type I muscle fibers are already recruited at lower work intensities during voluntary exercise (in cortically-stimulated muscle), and therefore ESA intensity does not increase with muscle work at higher work intensities (ATP usage activities), where predominantly type II fibers are recruited.

### Study limitations

Every computer model of a complex metabolic system can be at best only a certain approximation and simplification of the reality. Also the model of the skeletal muscle bioenergetic system used in the present study has some limitations.

The model does not describe any particular type of muscle fibers, but rather mixture of different fibers, and therefore constitutes a one-compartment model. On the other hand, it is compared just with ‘one-compartment’ experimental data, averaged over mixed muscle fiber population, such as the values of (pulmonary and muscle) $\dot{V}O_{2}$ (and, consequently, of τ_p_) as well as of PCr, P_i_, ATP and pH measured using the ^31^P MRS method.

A two-compartment model, distinguishing type I and type II muscle fibers, of the bioenergetic system of skeletal muscle was developed by Li and co-workers [51]. While this model generally reproduces well selected experimental data, these are essentially one-compartment data averaged over entire muscle containing type I and type II fibers. Therefore, the benefits from taking into account explicitly two compartments within this model seem limited.

The kinetics of the ‘additional’ ATP usage was simply assumed in the model. Nevertheless, the assumed linear increase of the ‘additional’ ATP usage in time gives a good agreement of computer simulations with experimental data [1].

The present theoretical study does not involve AMP deamination at very high exercise intensities. It was demonstrated in a previous simulation study that this process (when associated with the adenylate kinase-catalyzed reaction) constitutes in fact an additional (although minor) ATP source [34]. It was also shown that in the presence of intensive AMP deamination (+ adenylate kinase) ADP increases during the first ~2 min of exercise, but afterwards starts to decrease continuously with. This could account for the decrease in ADP with the relative ATP usage activity A_UT_ seen in Fig 8A and in Fig 2 for re-scaled experimental points.

Some parameter values, such as K_AUT_ were not directly extracted from experimental data, but assumed as ‘sufficiently small’ to give the saturating-type A_OX_-A_UT_ dependence.

However, these limitations do not seem to affect significantly the general conclusions formulated in the present theoretical study.

## Conclusions

Each-step activation (ESA) of oxidative phosphorylation (OXPHOS), NADH supply and glycolysis was proposed previously to be the main mechanism responsible for the regulation of the myocyte bioenergetic system during work transitions. The present theoretical study postulates that the regulation of OXPHOS, namely the A_OX_ (ESA intensity)-A_UT_ (relative ATP usage activity) dependence, is different in electrically-stimulated and cortically-stimulated skeletal muscle. The saturating-type A_OX_-A_UT_ dependence in the presence of the ‘additional’ ATP usage reproduces well the kinetic properties of the bioenergetic system of human skeletal muscle in voluntary constant-power exercise (cortical muscle stimulation). Computer simulations carried out assuming these system properties predict: 1. slow component of the $\dot{V}O_{2}$ on-kinetics in heavy / severe exercise; 2. non-linear, with a characteristic ‘change point’, $\dot{V}O_{2}$-A_UT_ (relative ATP usage activity related to power output) relationship determined 6 min after the onset of exercise; 3. linear PCr-A_UT_ relationship; 4. linear P_i_-A_UT_ relationship; 5. strongly non-linear ADP-A_UT_ relationship; 6. accelerating cytosol acidification above the critical ATP usage activity; 7. τ_p_ (characteristic transition time) of the principal phase of the muscle $\dot{V}O_{2}$ on-kinetics little dependent on A_UT_ (and thus on power output). All these system properties are encountered in experimental studies in voluntary constant-power exercise (cortically-stimulated muscle) in humans.

On the other hand, the power-type A_OX_ (ESA, each-step activation intensity)-A_UT_ (relative ATP usage activity) dependence in the absence of the ‘additional’ ATP usage describes well the bioenergetic system of electrically-stimulated rat skeletal muscle.

It is postulated that the regulation of OXPHOS is different in electrically- and cortically-stimulated (voluntary exercise) skeletal muscle. In the former, the A_OX_ (ESA, each-step activation intensity)-A_UT_ (relative ATP usage activity) dependence is power-type and the ‘additional’ ATP usage is absent. In the latter, the A_OX_-A_UT_ dependence is saturating-type and the ‘additional’ ATP usage is present. In electrically-stimulated muscle, all fiber types are recruited in parallel already at low ATP usage activities / work intensities. On the other hand, in cortically-stimulated muscle (voluntary exercise), type I fibers with high ESA intensity are stimulated at lower power output values, while type II fibers (especially type II b and IIx fibers) with low ESA intensity are recruited predominantly at higher power output values.
